# Supplementary material for: Modeling glioblastoma heterogeneity as a dynamic network of cell states
Source: Mol Syst Biol. 2021 Sep 16;17(9):e10105. doi: 10.15252/msb.202010105 (PMC8444284; doi:10.15252/msb.202010105)
Supplement: Supplementary file 6 — Source Data for Figure 5 [file MSB-17-e10105-s004.zip › Figure5A_sourcedata/GSEA_3071/nosigdb_stateIV.GseaPreranked.1622795164626/RICHARDS_ZHONG_NATURE_2018_OPC_UPREG.html]

Details for gene set RICHARDS\_ZHONG\_NATURE\_2018\_OPC\_UPREG[GSEA]

|  || Dataset | state53071 |
| Phenotype | NoPhenotypeAvailable |
| Upregulated in class | na\_pos |
| GeneSet | RICHARDS\_ZHONG\_NATURE\_2018\_OPC\_UPREG |
| Enrichment Score (ES) | 0.6619562 |
| Normalized Enrichment Score (NES) | 3.2138777 |
| Nominal p-value | 0.0 |
| FDR q-value | 0.0 |
| FWER p-Value | 0.0 |
Table: GSEA Results Summary

  

Fig 1: Enrichment plot: RICHARDS\_ZHONG\_NATURE\_2018\_OPC\_UPREG      
 Profile of the Running ES Score & Positions of GeneSet Members on the Rank Ordered List

  

| PROBE | GENE SYMBOL | GENE\_TITLE | RANK IN GENE LIST | RANK METRIC SCORE | RUNNING ES | CORE ENRICHMENT || 1 | PDGFRA |  |  | 1 | 2.924 | 0.0477 | Yes |
| 2 | PTPRZ1 |  |  | 10 | 2.539 | 0.0877 | Yes |
| 3 | PMP2 |  |  | 11 | 2.494 | 0.1286 | Yes |
| 4 | S100B |  |  | 12 | 2.461 | 0.1689 | Yes |
| 5 | GPM6B |  |  | 21 | 2.210 | 0.2035 | Yes |
| 6 | PTN |  |  | 25 | 2.140 | 0.2380 | Yes |
| 7 | SCRG1 |  |  | 28 | 2.056 | 0.2713 | Yes |
| 8 | LIMA1 |  |  | 58 | 1.805 | 0.2953 | Yes |
| 9 | SLC35F1 |  |  | 78 | 1.647 | 0.3186 | Yes |
| 10 | SPRY1 |  |  | 103 | 1.523 | 0.3390 | Yes |
| 11 | LSAMP |  |  | 130 | 1.454 | 0.3578 | Yes |
| 12 | BCHE |  |  | 155 | 1.362 | 0.3755 | Yes |
| 13 | DUSP6 |  |  | 156 | 1.359 | 0.3978 | Yes |
| 14 | GNG7 |  |  | 157 | 1.350 | 0.4199 | Yes |
| 15 | ETV5 |  |  | 174 | 1.305 | 0.4382 | Yes |
| 16 | SOX8 |  |  | 199 | 1.263 | 0.4543 | Yes |
| 17 | SOX6 |  |  | 207 | 1.244 | 0.4733 | Yes |
| 18 | CD82 |  |  | 223 | 1.202 | 0.4902 | Yes |
| 19 | NOVA1 |  |  | 234 | 1.181 | 0.5076 | Yes |
| 20 | SEMA5A |  |  | 247 | 1.153 | 0.5242 | Yes |
| 21 | PLAT |  |  | 281 | 1.070 | 0.5354 | Yes |
| 22 | CD9 |  |  | 288 | 1.062 | 0.5516 | Yes |
| 23 | SPRY4 |  |  | 304 | 1.030 | 0.5656 | Yes |
| 24 | ITGB8 |  |  | 307 | 1.027 | 0.5821 | Yes |
| 25 | DBI |  |  | 339 | 0.980 | 0.5922 | Yes |
| 26 | OLIG1 |  |  | 391 | 0.914 | 0.5974 | Yes |
| 27 | TRIB2 |  |  | 396 | 0.911 | 0.6115 | Yes |
| 28 | TTYH1 |  |  | 404 | 0.906 | 0.6250 | Yes |
| 29 | RFTN2 |  |  | 432 | 0.868 | 0.6341 | Yes |
| 30 | ETV1 |  |  | 488 | 0.824 | 0.6371 | Yes |
| 31 | PDE4B |  |  | 519 | 0.798 | 0.6444 | Yes |
| 32 | SCD5 |  |  | 555 | 0.759 | 0.6501 | Yes |
| 33 | SPATA6 |  |  | 559 | 0.756 | 0.6620 | Yes |
| 34 | PLEKHB1 |  |  | 648 | 0.692 | 0.6565 | No |
| 35 | ITM2C |  |  | 724 | 0.645 | 0.6527 | No |
| 36 | RAB31 |  |  | 864 | 0.574 | 0.6355 | No |
| 37 | SLITRK2 |  |  | 889 | 0.565 | 0.6402 | No |
| 38 | MT3 |  |  | 913 | 0.555 | 0.6449 | No |
| 39 | OLIG2 |  |  | 1129 | 0.486 | 0.6118 | No |
| 40 | GATM |  |  | 1148 | 0.481 | 0.6162 | No |
| 41 | TSC22D4 |  |  | 1176 | 0.472 | 0.6188 | No |
| 42 | TSPAN7 |  |  | 1181 | 0.471 | 0.6257 | No |
| 43 | ANGPTL2 |  |  | 1420 | 0.412 | 0.5870 | No |
| 44 | C2orf80 |  |  | 1447 | 0.403 | 0.5886 | No |
| 45 | EDNRB |  |  | 1549 | 0.378 | 0.5755 | No |
| 46 | CNP |  |  | 1575 | 0.371 | 0.5768 | No |
| 47 | LRRC4C |  |  | 1737 | 0.342 | 0.5516 | No |
| 48 | LMF1 |  |  | 1749 | 0.339 | 0.5551 | No |
| 49 | NXPH1 |  |  | 1950 | 0.306 | 0.5218 | No |
| 50 | SEZ6L |  |  | 2065 | 0.287 | 0.5048 | No |
| 51 | PON2 |  |  | 2177 | 0.271 | 0.4880 | No |
| 52 | B2M |  |  | 2349 | -0.252 | 0.4594 | No |
| 53 | RHOC |  |  | 4149 | -0.555 | 0.1247 | No |
| 54 | CST3 |  |  | 4577 | -0.723 | 0.0549 | No |
| 55 | ARL4A |  |  | 4695 | -0.791 | 0.0455 | No |
| 56 | ANXA5 |  |  | 4724 | -0.810 | 0.0534 | No |
| 57 | PSAT1 |  |  | 4867 | -0.932 | 0.0415 | No |
| 58 | EPB41L2 |  |  | 4974 | -1.051 | 0.0385 | No |
| 59 | ALCAM |  |  | 5118 | -1.327 | 0.0329 | No |
Table: GSEA details [plain text format]

  

Fig 2: RICHARDS\_ZHONG\_NATURE\_2018\_OPC\_UPREG: Random ES distribution      
 Gene set null distribution of ES for **RICHARDS\_ZHONG\_NATURE\_2018\_OPC\_UPREG**
